# Functional Traits Help Predict Post-Disturbance Demography of Tropical Trees

**DOI:** 10.1371/journal.pone.0105022

**Published:** 2014-09-16

**Authors:** Olivier Flores, Bruno Hérault, Matthieu Delcamp, Éric Garnier, Sylvie Gourlet-Fleury

**Affiliations:** 1 Cirad - Université de La Réunion, UMR PVBMT, 7 chemin de l'IRAT, Saint Pierre, France; 2 Cirad, UMR Ecologie des Forêts de Guyane, Kourou, France; 3 Cirad, UR B&SEF, Biens et Services des Ecosystèmes Forestiers tropicaux, Campus International de Baillarguet, TA C-105/D, Montpellier, France; 4 Centre d'Écologie Fonctionnelle et Évolutive, CNRS – UMR 5175, Montpellier, France; Institute of Botany, Czech Academy of Sciences, Czech Republic

## Abstract

How tropical tree species respond to disturbance is a central issue of forest ecology, conservation and resource management. We define a hierarchical model to investigate how functional traits measured in control plots relate to the population change rate and to demographic rates for recruitment and mortality after disturbance by logging operations. Population change and demographic rates were quantified on a 12-year period after disturbance and related to seven functional traits measured in control plots. The model was calibrated using a Bayesian Network approach on 53 species surveyed in permanent forest plots (37.5 ha) at Paracou in French Guiana. The network analysis allowed us to highlight both direct and indirect relationships among predictive variables. Overall, 89% of interspecific variability in the population change rate after disturbance were explained by the two demographic rates, the recruitment rate being the most explicative variable. Three direct drivers explained 45% of the variability in recruitment rates, including leaf phosphorus concentration, with a positive effect, and seed size and wood density with negative effects. Mortality rates were explained by interspecific variability in maximum diameter only (25%). Wood density, leaf nitrogen concentration, maximum diameter and seed size were not explained by variables in the analysis and thus appear as independent drivers of post-disturbance demography. Relationships between functional traits and demographic parameters were consistent with results found in undisturbed forests. Functional traits measured in control conditions can thus help predict the fate of tropical tree species after disturbance. Indirect relationships also suggest how different processes interact to mediate species demographic response.

## Introduction

**Figure 1:**
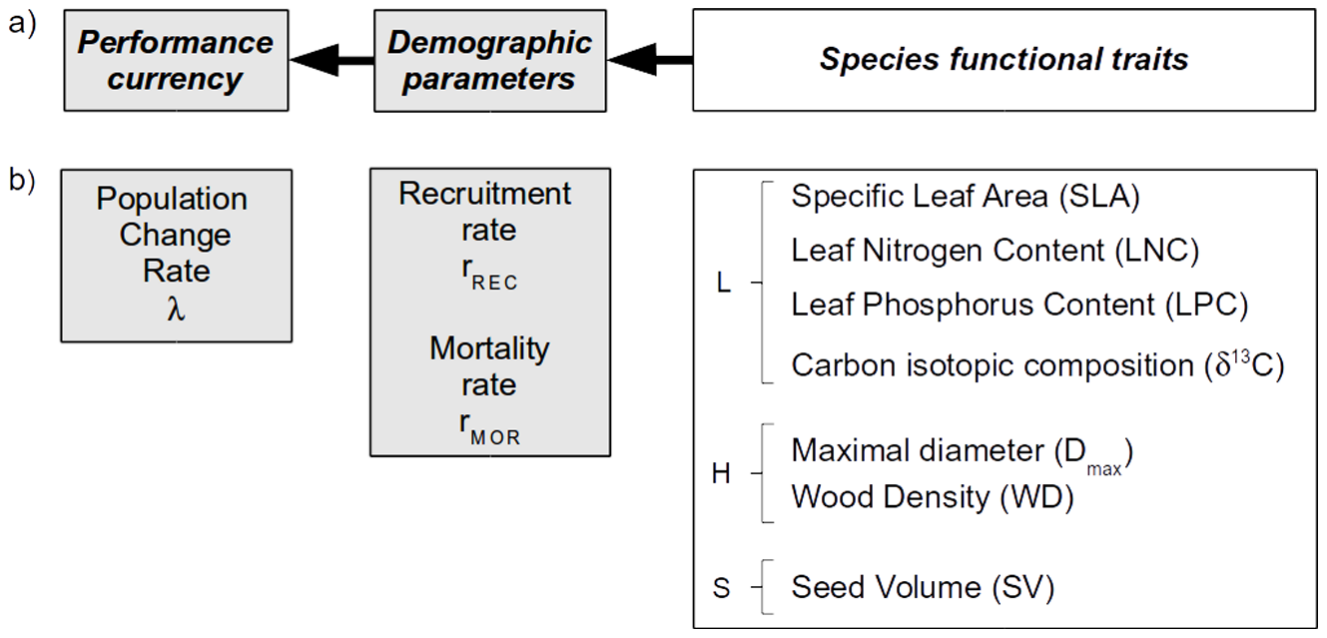
Ecological and data models. a) Hierarchical theoretical relationships between *Performance currency* [3] measured at population level and indicative of species performance, *Demographic parameters* measured at population level and indicative of population dynamics, and *Species functional traits* measured at individual level and indicative of species strategies [4]. b) Data model: variables used to quantify the components of the ecological model. Performance is evaluated by the population change rate, 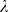; population dynamics is evaluated by recruitment and mortality rates; seven functional traits are considered in relation with the LHS scheme [9]. Gray and white boxes indicate variables measured in disturbed and control conditions, respectively.

Functional ecology assumes that biological functions scale up from the organismic level to higher organization levels [1]–[4]. At the individual level, biological functions can be investigated by means of functional traits which are morphological, physiological or phenological characteristics that influence individual fitness through growth, fecundity and survival [4]. In turn, the performance of all individuals in a population integrates into the population change rate as assessed in demographic studies, that is the outcome of the demographic processes of recruitment and mortality. Hence the relationships between the population change rate and functional traits are not direct but indirect through demographic parameters. Dependencies among population change, demographic processes and functional traits are widely acknowledged [3], [5], [6], but have rarely been integrated into a consistent model. Here, we evaluate an ecological model [7] connecting these variables in a three-level hierarchy (Figure 1), in order to identify key traits indicative of species response to disturbance in tropical forests. Although the model is general, key issues concern the nature of relevant traits and how they relate to demographic parameters.

Among key functional traits that capture most of the interspecific variation in plant strategies [8], and might thus drive species performance [3], Specific Leaf Area (SLA) stands as a proxy of mass-based maximum net photosynthesis, and ultimately relative growth rate [9], species size at maturity relates to investment in perennial structures and access to light [10], and seed mass differentiates species with respect to dispersal and survival in early life-stages [11]. These three key traits describe the LHS scheme of [9] and are indicative of species ecological strategies. Beyond these, we consider four other functional traits that relate to critical components of species response to disturbance. Wood density (WD) correlates with growth potential [12], resource allocation, and resistance to damage and embolism [5], [13]–[15]. Leaf nitrogen concentration (LNC) reflects the concentration of proteins involved in photosynthesis, and thus relates to net photosynthesis rate [16]. Leaf phosphorus concentration (LPC) also relates to photosynthetic capacity and ultimately growth [17]–[19]. Lastly, leaf carbon isotope discrimination (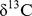) relates to water use efficiency [20], which may be critical in large forest gaps where sun exposure can cause drought.

In tropical forests, relationships between demographic processes and functional traits have been mostly investigated in plots in natural conditions [12] even if there are some recent attempts to link functional traits to disturbance tree responses using long-term plots from secondary forests [21], [22]. Species mortality rate usually decreases with increasing wood density [5], [14], adult stature [5], and seed size [5]. By contrast, evidence of correlates of recruitment rate is scarce in wild tree populations. Large seeds usually favor survival in early life-stages, but high recruitment rates are often associated with the pioneer syndrome of species producing small and numerous wind- or animal-dispersed seeds [23], [24], whereas recruitment efficiency, that is the recruitment rate adjusted for species abundance, decreases with increasing species maximum height [25], [26] or maximum diameter [27]. All together, these relationships demonstrate that functional traits can explain demographic parameters in undisturbed conditions to some extent, as presented in the model in Fig. 1.

In this paper, we hypothesize that traits measured in control conditions can explain, directly or indirectly the demographic response to disturbance. To test this hypothesis, we investigated the relationships between species traits measured in control conditions, and demographic parameters after disturbance due to logging operations. When investigating relationships between a given trait and a given demographical process, correlation may reflect a direct link due to a causative relationship, or an indirect link because the two variables are under the influence of a third one. Hence, some traits may have direct effects on species demography in response to disturbance, while others may have an indirect influence. Such trait-based approach allows one to better understand the underlying mechanisms of why some are successful after disturbance, and others not. It may also initiate a step forward into predicting the future of tree species communities undergoing disturbance over large areas more easily than by settling permanent sample plots. We specifically address the following questions: *(i)* how are population changes in response to disturbance partitioned with regard to recruitment and mortality ? *(ii)* how are demographic process rates controlled by functional traits ? and *(iii)* which traits directly *vs* indirectly influence the demographic rates ?

The purpose of this paper is to address these questions simultaneously in a consistent ecological model. Regression and variable selection methods can help address the two first questions. The third question however supposes a hierarchical structure among variables, with traits having either direct or indirect influence on demographic parameters. We used the Bayesian Network (BN) framework to investigate the hierarchical structure of the ecological model presented in Fig. 1. Other approaches, such as the Structural Equation Modelling framework also allow to quantify the likelihood of a network of relationships given data. However, a key aspect of the BN approach is that the structure of the Direct Acyclic Graph (DAG) that represents the network is not given *a priori* but can be inferred from the data. We applied the BN methodology to investigate the demographic response of fifty-three tropical tree species to disturbance. We used long term survey data of population dynamics in logged-over permanent sample plots located in French Guiana [27] to quantify species population change rates as well as demographic parameters. These data were completed by species-specific attributes collected for the seven functional traits. After investigating correlative patterns, we present the BN framework and apply it to the questions raised above. We then discuss the inferred relationships and whether they are similar to those observed in undisturbed forests.

## Materials and Methods

### Study site and period

The study was conducted at the Paracou Experimental site (5°18′N, 52°53′W) in a lowland *terra firme* rainforest of French Guiana dominated by the *Caesalpiniaceae*, *Lecythidaceae*, *Chrysobalanaceae* and *Sapotaceae* families. The site is located on private land and rented by the research institute (CIRAD). CIRAD is given full permission by the owner to conduct studies on the land, including the one presented in this manuscript. No specific permission was required for the activities conducted during the study (*in situ* measurements and collections). Field collections did not involve any endangered or protected species.

The climate of the area is equatorial with a dry season from mid-August to mid-November and a rainy season, often interrupted by a dry period in March. The mean annual rainfall and temperature are 3040 mm and 26°C. Soils are mostly shallow ferralitic and developed on schists and sandstones that form small elliptic hills.

The experimental setting of the site consists in fifteen 9-ha permanent sample plots (PSP), 9 of which were submitted to three logging treatments of increasing intensity (T1, T2, T3), while 6 remained control plots in natural conditions (T0). Each 9-ha plot contains a core zone of 6.25 ha surrounded by a 25 m wide buffer zone. In 1984, all trees with diameter at breast height (DBH) 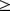 10 cm were localized in the 250
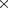
250 m core zone of each plot and botanically identified. Since then, all plots have been surveyed annually to evaluate individual growth, as well as recruitment and mortality. Logging operations were conducted during two years between 1986 and 1988 at the scale of the 9-ha plots. The treatments involved selective harvesting for timber (T1, T2, T3: 10 commercial trees.ha^−1^ DBH 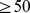 or 60 cm), and fuelwood (T3: 30 non-commercial trees.ha^−1^ with DBH between 40 and 50 cm), and additional thinning of non-commercial trees by poison-girdling (T2: 30 trees.ha^−1^ DBH 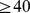 cm, T3: 20 trees.ha^−1^ DBH 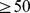 cm; [28]).

In this study, we focus on the consequences of disturbance on population dynamics in terms of indirect effects. Direct effects of selective logging and poison-girdling include tree damage and destabilization by neighbours loss. These effects were materialized by the death of wounded trees up to four years after operations stopped. Here, we are interested in the response of species to the renewed availability of resources (space, light and soil) after disturbance. In order to eliminate the direct effects of the treatments, we thus calculated the population change rates and demographic parameters starting four years after the end of logging operations, over the period 1992–2003. We considered all trees 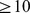 cm DBH occurring in the six most heavily disturbed plots, *i.e.* in treatments T2 and T3.

### Focal Species

Fifty-three species were selected for this study; they were well-documented in floras, unambiguously identified by field workers, and abundant enough to supply relevant data (see Appendix S1). They represented 28 families with a dominance of *Caesalpiniaceae* (13 species), *Sapotaceae* (4), *Euphorbiaceae* (4), *Clusiaceae* (4), *Lecythidaceae* (3) and *Myristicaceae* (3), which reflects the local composition of the forest. The studied species cover a large range of ecological strategies from small and medium-sized heavy shade-tolerant species to small light-demanding species and large light-demanding canopy species, including three pioneer species. In order to characterize these strategies, we estimated species growth potential in control conditions. We measured growth potential (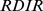 for relative diameter increase rate) as the 95^*th*^ percentile of the distribution of change rate in relative diameter increment
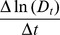




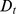
 being the DBH at year 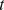, and 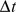 taken here between 2000 and 2003 [27]. Note that, because there is no direct link between population change rates and species 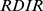
s, we chose to not include the latter in the presented bayesian network analysis. However, we reported their values in table S1 in Appendix S1 and performed the network analysis including 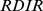 for comparison purposes (Figure S1).

### Demographic Parameters and Population Change Rate

We calculated the population change rate over the study period, 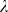, as
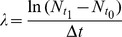
where 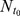 and 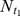 are the number of living trees at the beginning (1992) and at the end (2003) of the study period of length 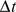. In order to take advantage of the yearly surveys, we calculated recruitment, defined as ingrowth over 10 cm DBH threshold (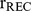), and mortality (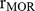) rates following [29] as
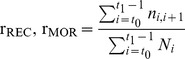
where 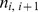 is the number of trees respectively recruited (*i.e.* reaching 10 cm DBH) or dying between years 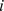 and 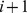, and 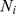 is the number of living trees in year 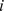. We preferred this approach to estimate demographic parameters because estimations based on the raw comparison of an initial and a final survey [30] cannot account for trees both recruiting and dying during the study period. We calculated mortality rates excluding trees that had been wounded or poisoned during silvicultural operations in order to avoid direct effects of disturbance and capture intrinsic specific responses to disturbance. Note that although populations change in number as a result of the demographic processes of recruitment and mortality, the definitions of the corresponding rates do not lead to 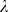 being a simple combination of the demographic parameters.

### Functional Traits

Leaf traits were measured on leaves collected in three undisturbed control plots of Paracou following standardized protocols [31]. As far as possible, local measurements in control plots have been preferred to values from global trait databases in order to obtain locally accurate estimates of the interspecific trait variation [32]. Leaves were taken in fully sunlit positions, except for three species of small size (*Iryanthera hostmannii, Oxandra asbeckii* and *Sandwithia guianensis*). Ten leaves in total were collected on 3 different trees of each species. Analyses were performed on complete leaves, with petiole and rachis, except when rachises were too hard to be ground (*Carapa procera*, *Jacaranda copaia*, *Schefflera decaphylla*, *Simaba cedron*).

We measured the specific leaf area (SLA) as the one-sided area of fresh leaves divided by the oven-dried mass. Leaf nitrogen and phosphorus concentrations (LNC and LPC, the total amount of N and P per unit of leaf dry mass) were measured respectively by gas chromatography performed on a Carlo Erba CHN-OS elemental analyser, and by the molybdate-blue method, using ascorbic acid as a reductant. Leaf carbon isotope composition (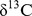) was measured using a Finnigan DELTA S isotopic ratio-mass spectrometer.

As an estimate of species potential size, we considered the six control plots of the site in 2003 for the reference distribution of diameters and calculated the maximum diameter (D_*m*_) as the 95^*th*^ percentile of the left-truncated distribution, that is all diameters above the threshold of 10% of the species absolute maximum, as recommended in [33]. We estimated seed size as the ellipsoidal volume calculated from measured dimensions:
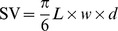




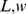
 and 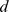 being respectively the length, the width and the depth of a seed. Seed mass was highly correlated to seed volume for a subset of species, but we preferred the latter trait because it was available for all studied species. Finally, wood density (WD), the ratio between mass and volume of a piece of wood measured at 12% moisture content was compiled from a local database and literature. Trait data for the focal species are presented in Table S2 in Appendix S1.

### Bayesian Network Analysis

In graph theory, relationships within a set of studied variables 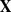 can be represented by a Directed Acyclic Graph (DAG). A DAG consists in a set of nodes (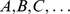) and directed edges between them representing the variables and the dependencies among them respectively: 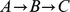 for instance. In the Bayesian Network (BN) framework [34], [35], a statistical model of these dependencies is entirely defined by the DAG and an associated probability that measures the likelihood of the represented dependencies given the data (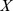).

In a DAG, the parents of a node 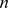 are the nodes that connect directly onto 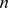, noted 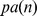. When conditioned on its parents, a particular node is by definition independent of all other variables that are not among the parents. Within each *parents*–*child* cluster, the dependency of the child node on its parents is thus described by a conditional probability distribution, 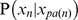, where 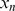 is the variable associated with node 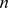. The set of distributions for all clusters in the DAG allows to define the likelihood of the DAG given the data
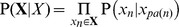



Specifically, the conditional distributions are modelled as Gaussian linear models [36]:
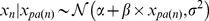
(1)where 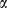 and 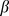 are regression coefficients and 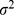 is the conditional variance. We thus log-transformed some variables in the analysis (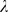, 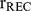, 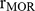
and SV) to ensure that their distributions approached normality (Table S2 & S3 in Appendix S1).

Conditionnal dependence or independence among the studied variables can be directly read from a DAG. In the simple example 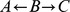, both nodes 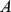 and 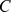 depend on 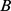, so that 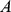 and 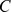 are dependent when conditioned on 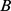, *i.e.* when one considers 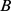 as fixed [36]. In the example 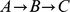, node 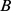 depends on 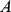, and 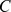 depends on both 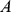 (indirectly) and 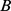 (directly), but 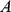 and 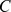 are independent conditionally on 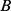, that is 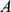 and 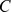 can vary independently when 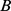 is fixed. Finally, in the example 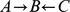, node 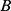 depends on both 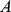 and 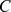, but 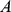 and 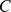 are independent because no edge exists between them. In the present study, the DAG has an inherent hierarchical structure given by the considered ecological model (Figure 1). In order to impose the sense of some of the arrows, relationships were specifically banned from the network [36]: we did not allow arrows directed from 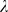 to demographic parameters or traits, neither from demographic parameters to traits, or from traits to 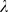.

We used a score-based approach to infer the structure of the DAG from the observed data: the algorithm goes through the space of possible networks, scores candidates based on the likelihood and finds the best scoring network [36]. We used a weakly informative prior for the joint probability, thus assuming weak *a priori* knowledge about the network structure. After a likely network was found, we performed an intensive heuristic search, which consists in perturbing the DAG a large number of times and performing multiple random restarts using the new network. This approach insures that a global maximum score has been reached.

We then performed complementary tests to ensure that the inferred multivariate relationships implied by the network were statistically valid. We first tested the overall structure of the network using Fisher's C test as described by [37]. We then tested all inferred relationships implied by the DAG structure ([37], [38], see Table S4 in Appendix S1) using partial correlation coefficients
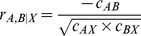
where 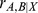 measures the correlation between variables 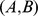 conditionally on variables in 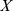, and 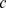 is the correlation between corresponding variables. We used Pearson's correlation coefficient to ensure consistency with the linearity hypothesis of the BN approach, as well as Spearman's correlation coefficient. All inferred conditional independencies were eventually verified. Inferred conditional dependencies present in the calibrated network that were not significant with regards to both coefficients (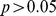) were removed from the analysis to ensure a conservative approach. P-values were corrected for multiple testing. All analyses were performed with R [39] using the *deal* [36] and *ggm* packages for BN and DAG analysis.

## Results

**Figure 2:**
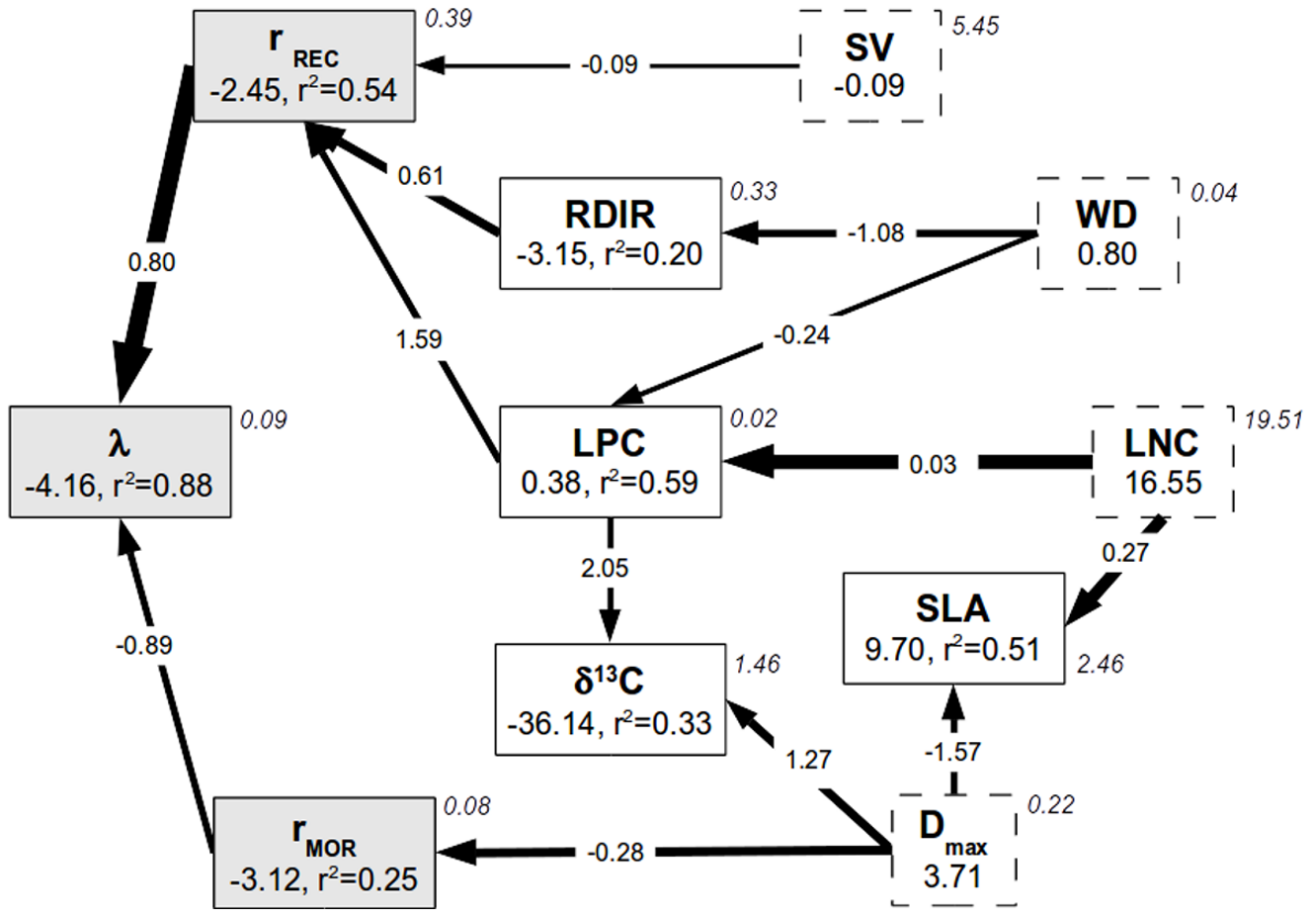
Results of the Bayesian Network analysis. Directed Acyclic Graph (DAG) showing the network of relationships between the population growth rate (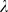), demographic parameters (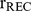, recruitment rate, 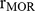, mortality rate) and functional traits (see main text for labels). Grey (white) indicates variables measured after disturbance (in control conditions). Dashed boxes indicate variables having no parent (explicative variable) in the network: SV, WD, LNC and 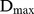. The figure represents a statistical summary of the inferred relationships. Numbers indicate the posterior estimates of the model parameters: the intercept 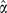 and the proportion of variance explained by the parents (r^2^; see relation 1) are given in boxes, the regression coefficients (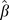) are shown on arrows. Arrow width is proportional to the corresponding standardized regression coefficient. Italic numbers are estimates of the residual variance (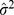; see relation 1, to be compared with raw variances in Table S3 in Appendix S1).

At the beginning of the study period, population size ranged from 19 individuals, in *Balizia pedicellaris* (Fabaceae) and *Schefflera decaphylla* (Araliaceae) to 1330 in *Lecythis persistens* (Lecythidaceae) for an average population size of 185.2 individuals (s.d.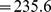, see Table S1 in Appendix S1). Population change rate over the study period varied from -0.01 (*Pogonophora schomburgkiana* Euphorbiaceae) to 0.25 (*Schefflera decaphylla*), while recruitment and mortality rates respectively varied from 0.4% (*Tapura capitulifera* Dichapetalaceae) to 15.1% (*Schefflera decaphylla*) and from 0 (*Platonia insignis* Clusiaceae) to 2.4% (*Pogonophora schomburgkiana*, Table S1 in Appendix S1). The BN analysis evidenced direct and indirect relationships among variables in a network supporting the ecological model in Figure 1. Population change rate, 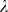, directly depended on the demographic parameters (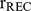,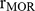) by construction. Overall, 88% of its variance were explained by those parameters, 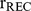 being the most influential variable (see arrow width in Figure 2) compared to 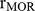.

The recruitment rate 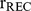 was directly related to three functional traits that together explained 45% of its variance (Figure 2): LPC had a positive effect, whereas wood density (WD), and seed volume (SV) had negative effects on 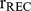. When species growth potential (RDIR) was included in the analysis, it appeared in a direct relationship with 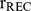 and the proportion of explained variance raised to 54% (Figure S1). The mortality rate was explained by a single functional trait, species maximum diameter (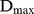, 25%). Among functional traits, LPC was explained by WD and LNC (59%). SLA was in turn informed by LNC and 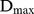 (51%). Lastly, carbon isotope discrimination, 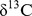, was informed by LPC and 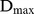 (33%). The relationships between functional traits and demographic parameters were only slightly modified when we included species growth potential in the analysis. The only difference in the structure of the DAG was that the relationship between WD and 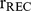 became indirect and mediated by species growth (Figure S1): in this model, the recruitment rate was then independent of wood density when conditioned on, *i.e.* for given, growth potential and leaf phosphorus concentration.

**Figure 3:**
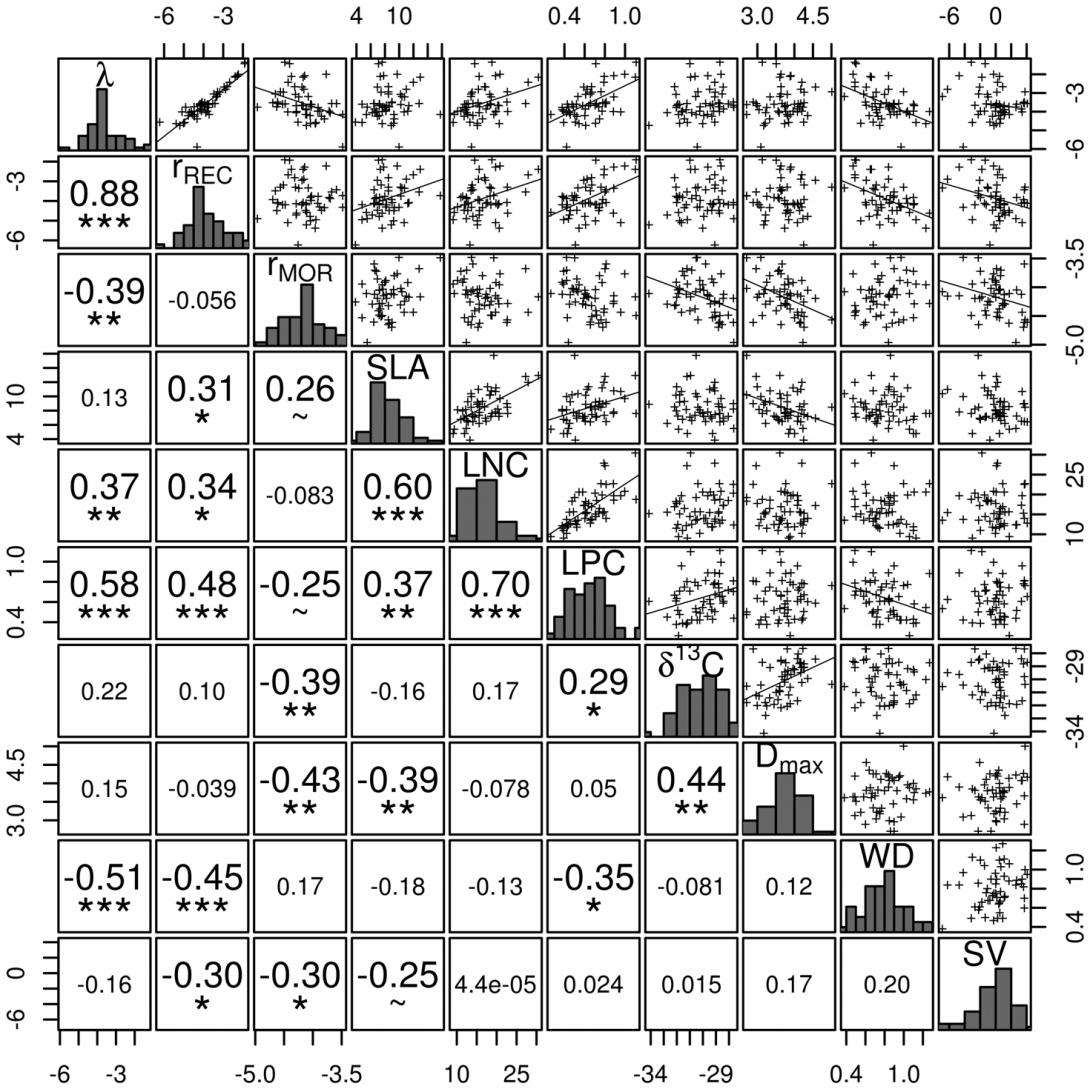
Correlation analysis. Relationships between population change rate (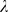) and demographic parameters (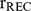, 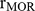) after disturbance and functional traits measured in control conditions (see main text for labels definition). Panels show respectively variables distribution in the diagonal, pairwise connections with lines showing linear regressions in the top triangle, and Spearman correlation coefficients on rank with tests results in the lower triangle. Large numbers indicate significant correlations with p-values as follows: 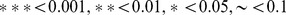. 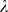, 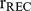, 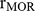, and SV were log-transformed.

The network analysis highlighted four variables without parents (exogenous variables; Figure 2): SV, WD, LNC and 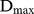. SV appeared on a single path to 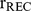, while WD, LNC and 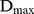 appeared on forks, each being directly related to two descending variables. The positions of these exogenous variables, and assuming that errors on them are independent, indicate that these variables are independent pairwise (as shown in Fig. 3). Moreover their positions as exogeneous variables highlight their influence on other relationships. For instance, the position of 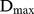 as unique parent for 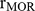 indicates that the mortality rate was independent from the other exogeneous variables when conditioned on maximum size (Table S4 in Appendix S1). Hence, the correlation observed between 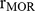 and SV (Figure 3) was mediated by 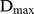: the two variables appeared independent when conditioned on maximum size (Table S4 in Appendix S1): for a given maximum size, the mortality rate is independent of seed volume.

The DAG allows us to highlight independences involving not only exogenous, but also endogenous variables (see Table S4 in Appendix S1 for a list of relationships). For instance, pairwise correlation shows that 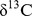 correlates negatively to the mortality rate (Fig. 3). However, according to the DAG, the two variables are not in a direct relationship and they are in fact independent when conditioned on their parents 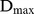 and LPC (Table S4 in Appendix S1). This finding can be interpreted as mortality rate being independent from carbon isotope composition in species having similar maximum diameter and leaf phosphorus concentration. Regarding functional traits, the same reasoning applies to the covariation between LPC and SLA, that are independent when conditioned on WD, LNC and 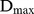, and SLA and 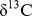, independent when conditioned on LNC, 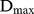 and LPC (Table S4 in Appendix S1). We note that these findings are contextual, they derive from the statistical properties of the studied dataset and may not be straightforward to interpret biologically.

## Discussion

Our analysis demonstrates that functional traits measured in control plots can explain species demography assessed after disturbance. We highlighted both direct and indirect relationships between population change rate, demographic parameters and functional traits of tropical trees by taking advantage of the statistical dependencies among the variables to find the most explicative relationships of the hierarchy proposed in a simple ecological model (Figure 1).

### Population Change Rate and Demographic Parameters

After disturbance, recruitment critically controlled species demography, while mortality had revealed lower influence (Figure 2). Disturbance by logging induced instantaneous demographic imbalance in tree populations, because they were either directly eliminated by selective logging or poison-girdling, or indirectly killed by falling trees or following disturbance-induced stresses, implying that most of the final tree populations however recovered from reduced numbers [28]. This has resulted in increased recruitment [27], as well as increased growth after disturbance [40] when compared to populations in control plots. Regarding mortality, logging effects were more contrasted when compared to control conditions, with decreasing mortality rates observed in heliophilous species and increasing rates observed in shade-tolerant species, which indicated a negative effect of open canopy conditions on survival in the latter (see Table S1 in Appendix S1). These findings are consistent with previously documented disturbance effects on dynamics in logged-over stands [41]: dipterocarp forests showed higher recruitment rates in treated plots compared to control plots several years after mortality rates had returned to pre-disturbance levels. In forests of Venezuela, process-based simulations showed that ingrowth dominated over mortality after disturbance, leading to positive demographic imbalance [42]. We note that the time lag between logging operations and the study period, the length of the study period and the size threshold used to sample populations all determine how populations were sampled and may have consequences on the patterns highlighted here. Recruits come from germinated seeds or were present as advanced regeneration that has grown up to the threshold before the end of the study period. Because of their very low inherent growth rate, it is likely that recruitment in the more shade-tolerant species should come from advanced regeneration. A longer period of censuses would allow us to test the hypotheses of (i) a delayed and higher recruitment rates for shade-tolerant species together with (ii) a concomitant decrease in recruitment of light-demanding, and usually small-seeded species.

### Drivers of Demographic Parameters

About half of the interspecific variability in the recruitment rate was best explained by three functional traits, including leaf phosphorus concentration, wood density and seed size (Figure 2). In disturbed stands of the site, we know that a positive relationship exists between growth and recruitment rates [27]. Thus the revealed relationships between functional traits such as leaf phosphorus concentration and recruitment rate could be mediated by species growth potential. Phosphorus is a major component of plant growth [43], as it is present in nucleotides implied in metabolic pathways (e.g. adenosine triphosphate, ATP). Under well-developed vegetation and on ageing soils, weathering and biological mobilization decrease phosphorus availability, which may in turn limit plant growth and development [44], [45]. Nitrogen is also known to limit tree growth [43], however N:P ratios observed here revealed high values, from 16.9 to 38.5 (26.2 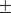
5.4), which may indicate limitation by P, combined limitation by both P and N or even N limitation [45]. Recent evidence however confirmed that phosphorus rather than nitrogen controled the productivity of Amazonian forests [46]. Here, our network analysis evidenced the central role of phosphorus in the system, suggesting that species best at mobilizing available phosphorus also better recruited in response to disturbance. Unexplained variability in recruitment rates proceeds from unobserved causes that are sources of variation in juveniles survival and growth, it relates to stochasticity in the effects of abiotic and biotic factors on these processes.

Wood density negatively influenced recruitment rate, which is consistent with the well-known negative correlation between wood density and growth [15], [33], [47]–[49]. High wood density implies high stem construction costs, mechanical stability and resistance to cavitation [14], [50], [51], as well as low photosynthetic rates [52]. By contrast, low wood density is associated with efficient resource acquisition, which allows species to reform population numbers rapidly after disturbance. At individual scale, wood density is negatively linked to the tree ability to accelerate its growth during early life stages [53], that is light-wooded species have the best potential to accelerate their growth at intermediate sizes in suitable conditions, resulting in a hump-shaped growth trajectory. On the contrary, heavy-wooded species with constrained construction potential generally show slow [12], but relatively constant, growth patterns [53].

Seed volume negatively influenced recruitment directly, suggesting that regeneration processes were involved in species response to disturbance: high disturbance intensity tended to preferentially favor small-seeded species. However, as found elsewhere, we note that correlations between demographic parameters and seed volume were weak [5]. In undisturbed tropical forests, seed size was negatively correlated to mortality [5]. Although not causally linked to mortality of large trees, this correlation can be interpreted as a consequence of the adaptive trade-off between tolerance to low resource supply and growth [11], [23]. Large seeds often occur in species with attributes related to shade-tolerance, whereas pioneer and light-demanding species disperse overall smaller and more numerous seeds [54].

Previous studies found that mortality rates were negatively correlated with wood density in undisturbed tropical forests [5], . In our case, wood density was unrelated to mortality rate measured after disturbance. We suspect that logging-induced disturbance has blurred connections between the mortality rate and wood density. During the study period, light-demanding species, which are also light-wooded, survived better in disturbed than in control conditions (Figure 2) due to higher light availability [27], while disturbance had lower effects on the mortality rate of more shade-tolerant species. It also appears that correlations between wood density and demographic parameters might be stronger when considered over larger spatial scales (but see [6], [56]) leading to wider variability in wood density in relation with, for instance, variation in soil fertility [57], [58].

Species of high stature showed significantly lower mortality rates than smaller ones, consistently with results observed in undisturbed forests [27], [56]. We suspect that disturbance reinforced this relationship in logged-over plots: species of small stature are often shade-tolerant and, although some of them benefited from canopy opening [40], the majority probably suffered from sun exposure. In logged forests, gap creation increases lateral lighting and temperature along the vegetation profile. Such conditions have also likely impacted species differentially according to their ability to optimize water use. Interestingly, mortality also varied with enrichment in heavy carbon isotope, 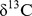. In sunlit leaves, 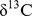 relates positively to water use efficiency (WUE; [59]), which measures the ratio between biomass gain through 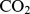 assimilation and water loss through evaporation. At Paracou forest site, water availability is the main climatic driver of tree growth [60]. It has been shown that tall tree species preferentially extract water from layers below 100 cm depth while shorter tree species show broader variations in the depth of water uptake [61]. Here, mortality decreased in species with increasing values of carbon isotope composition (higher WUE), but the correlation was indirect: the relationship appeared mediated by species stature and leaf phosphorus concentration. For a given maximum size, and for species with similar LPC, susceptibility to drought as indicated by carbon isotope composition, was not found to influence tree mortality.

Our network analysis evidenced the role of four functional traits as independent sources of interspecific variability. More specifically, leaf nitrogen concentration, wood density, maximum diameter and seed volume appeared as exogeneous variables, that is, variables not explained by other variables considered in the analysis. This finding constitutes an indirect validation of the leaf-height-seed (LHS) scheme which considers three major independent axes of variation in plant ecological strategies [9]. Among those four exogeneous variables, the proxy for the “leaf axis” of the LHS was leaf nitrogen concentration. Its relationships with the demographic parameters however were weak for recruitment and only marginal for mortality. Furthermore, SLA correlated better with LNC than LPC, LNC was more strongly correlated to LPC than SLA, and LPC appeared more strongly correlated to recruitment than LNC. Altogether, these relationships resulted in LNC acting as an independent axis of variation, LPC being a direct driver of recruitment and SLA having no direct influence on species response to disturbance. Wood density and maximum diameter characterized two different aspects of the “height” axis [12] in relation with resource allocation and conservation (WD) and resource acquisition (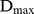). Eventually, seed volume independently positioned species along the seed axis of plant ecological strategies.

### Relationships among Functional Traits

We found close positive associations among three major traits involved in the so-called “leaf economics spectrum” (LES; [16]): SLA, LNC and LPC. These associations are indicative of coordination among the different processes leading to CO2 fixation by leaves, as SLA relates to light capture, LNC to carboxylation capacity, and LPC to energy transfer during photosynthesis. High values of these three traits lead to high rates of photosynthesis and respiration [16], [62].

The relationships between leaf carbon isotope composition, 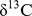 and the other traits of the LES are not straightforward to interpret. Relationships with leaf nutrients have not been found to be consistent across studies [63]–[65]. As an integrative proxy of WUE, 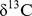 is strongly driven by stomatal conductance and maximum photosynthetic rate. Here, leaf 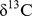 was found to be positively related to LPC (directly), but not to LNC, which may indicate a control of WUE by species photosynthetic activity rather than water flows. If one assumes low variance in stomatal conductance across species, these findings suggests that higher LPC implies higher 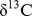 because of lower concentration of CO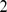 in leaves due to higher photosynthetic rates [66]. Such a relationship was not observed with nitrogen, which also suggests limitation of photosynthesis by P rather than N.

A low water use efficiency (low leaf 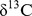 values) appears to be often related to a high SLA [63]–[65], [67], [68], but the lack of a relationship with SLA has already been found elsewhere [69], [70]. Clearly, a better understanding of how leaf 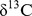 relates to traits of the LES is required, which implies a thorough analysis of how carbon and water economies are coordinated within the leaf. We also found that leaf 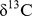 increased with species maximum diameter, a surrogate of maximum height. This pattern has been reported elsewhere both within [71], [72] and across species [20]. Gravity and xylem path length resistance induce a reduction in water potential at tree tops with increasing species height. This reduction is usually compensated for by decreased stomatal conductance leading to 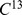-enrichment and increasing 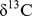 [72], [73].

## Conclusions

The present study reveals the potential of functional traits measured following standard protocols to explain and predict species performance in response to disturbance and logging operations in particular. Regarding recruitment, 45% of the variability in the process rate across species could be explained by three traits only, WD, SV and LPC, whereas for mortality one trait only, 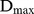, significantly explained the process rate up to 25%. These results are consistent with other studies considering functional traits as drivers of demographic processes (*e.g.* [6]). The discrepancy between the two processes partly results from the higher part of stochasticity in mortality in tropical forests. We used a novel approach to unravel indirect relationships showing a hierarchy of traits being more or less closely related to demographic parameters at population level. However, we acknowledge that the modelling framework used here did not allow interactions among traits to drive the demographic parameter values, although interspecific variation in demographical rates in nature is shaped by syndromes of functional traits [74], [75]. Interestingly four traits among those tested were independent of all others and therefore form a minimal set of predictors of species response to disturbance. LPC was however a better predictor of recruitment rates than LNC. These results evidence that local databases of functional traits can help understand and predict the response of forest communities to anthropogenic disturbance (see also [76]). Understanding tropical forests response to disturbance, including anthropic, is an important challenge to scientists which ideally requires long-term data collected in permanent plots with different disturbance regimes. These plots are often costly to manage, and cannot sample each particular “site 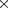 forest type” combination neither provide reliable estimates of demographic parameters for every tree species when many are scarce. One alternative to which this study contributes is to use biological proxies collected in natural conditions and used to anticipate species response to disturbance.

## Supporting Information

Figure S1 **Bayesian Network resulting from the addition of species growth amongst the set of studied variables.**
(TIF)

Appendix S1 **Tables S1-S4.** Table S1. Demographic and dynamics information on the 53 tropical tree species of the study. Table S2. Functional traits data for the tree species of the study. Table S3. Statistical summary of variables distributions and transformations. Table S4. Conditional independences derived from the DAG presented in main text.
(PDF)

## References

[1] Chapin F III, Zavaleta E, Eviner V, Naylor R, Vitousek P, et al (2000) Consequences of changing biodiversity. Nature 405: 234–242. 10821284 10.1038/35012241

[2] Grime J (2001) Plant strategies, vegetation processes, and ecosystem properties. John Wiley and Sons

[3] McGill BJ, Enquist BJ, Weiher E, Westoby M (2006) Rebuilding community ecology from functional traits. Trends in Ecology and Evolution 21: 178–185. 16701083 10.1016/j.tree.2006.02.002

[4] Violle C, Navas ML, Vile D, Kazakou E, Fortunel C, et al (2007) Let the concept of trait be functional!. Oikos 116: 882–892.

[5] Poorter L, Wright SJ, Paz H, Ackerly DD, Condit R, et al (2008) Are functional traits good predictors of demographic rates ? Evidence from five neotropical forests. Ecology 89: 1908–1920. 18705377 10.1890/07-0207.1

[6] Wright SJ, Kitajima K, Kraft NJ, Reich PB, Wright IJ, et al (2010) Functional traits and the growth-mortality trade-off in tropical trees. Ecology 91: 3664–3674. 21302837 10.1890/09-2335.1

[7] Austin M (2002) Spatial prediction of species distribution: an interface between ecological theory and statistical modelling. Ecological Modelling 157: 101–118.

[8] Westoby M, Falster D, Moles A, Vesk P, Wright I (2002) Plant ecological strategies: some leading dimensions of variation between species. Annual Review of Ecology and Systematics 33: 125–159.

[9] Westoby M (1998) A leaf-height-seed (LHS) plant ecology strategy scheme. Plant Soil 199: 213–227.

[10] Falster DS, Westoby M (2005) Tradeoffs between height growth rate, stem persistence and maximum height among plant species in a post-fire succession. Oikos 111: 57–66.

[11] Leishman MR, Wright IJ, Moles AT, Westoby M (2000) The evolutionary ecology of seed size, CAB International Press, chapter 2. Ed.2. pp. 31–57.

[12] Rüger N, Wirth C, Wright SJ, Condit R (2012) Functional traits explain light and size response of growth rates in tropical tree species. Ecology 93: 2626–2636. 23431593 10.1890/12-0622.1

[13] Falster D, Westoby M (2005) Alternative height strategies among 45 dicot rain forest species from tropical Queensland, Australia. Journal of Ecology 18: 337–343.

[14] King DA, Davies SJ, Noor NSM (2006) Growth and mortality are related to adult tree size in a malaysian mixed dipterocarp forest. Forest Ecology and Management 223: 152–158.

[15] Chave J, Coomes D, Jansen S, Lewis SL, Swenson NG, et al (2009) Towards a worldwide wood economics spectrum. Ecology Letters 12: 351–366. 19243406 10.1111/j.1461-0248.2009.01285.x

[16] Wright I, Reich P, Westoby M, Ackerly D, Baruch Z, et al (2004) The worldwide leaf economics spectrum. Nature 428: 821–827. 15103368 10.1038/nature02403

[17] Wright IJ, Reich PB, Westoby M (2001) Strategy shifts in leaf physiology, structure and nutrient content between species of high- and low-rainfall and high- and low-nutrient habitats. Functional Ecology 15: 423–434.

[18] Niinemets U, Kull K (2003) Leaf structure vs. nutrient relationships vary with soil conditions in temperate shrubs and trees. Acta Oecologica 24: 209–219.

[19] Reich PB, Oleksyn J (2004) Global patterns of plant leaf n and p in relation to temperature and latitude. Proceedings of the National Academy of Sciences 101: 11001–11006. 10.1073/pnas.0403588101 PMC503733 15213326

[20] Bonal D, Sabatier D, Montpied P, Tremeaux D, Guehl J (2000) Interspecific variability of delta13c among trees in rainforests of french guiana: functional groups and canopy integration. Oecologia 124: 454–468. 28308785 10.1007/PL00008871

[21] Carreño-Rocabado G, Peña-Claros M, Bongers F, Alarcón A, Licona JC, et al (2012) Effects of disturbance intensity on species and functional diversity in a tropical forest. Journal of Ecology 100: 1453–1463.

[22] Lasky JR, Uriarte M, Boukili VK, Erickson DL, John Kress W, et al. (2014) The relationship between tree biodiversity and biomass dynamics changes with tropical forest succession. Ecology Letters : n/a–n/a. 10.1111/ele.12322 24986005

[23] Moles AT, Westoby M (2006) Seed size and plant strategy across the whole life cycle. Oikos 113: 91–105.

[24] Baraloto C, Forget PM, Goldberg DE (2005) Seed mass, seedling size and neotropical tree seedling establishment. Journal of Ecology 93: 1156–1166.

[25] Kohyama T, Suzuki E, Partomihardjo T, Yamada T, Kubo T (2003) Tree species differentiation in growth, recruitment and allometry in relation to maximum height in a bornean mixed dipterocarp forest. Journal of Ecology 91: 797–806.

[26] King D, Wright S, Connell J (2006) The contribution of interspecific variation in maximum tree height to tropical and temperate diversity. Journal of Tropical Ecology 22: 11–24.

[27] Delcamp M, Gourlet-Fleury S, Flores O, Garnier E (2008) Can functional classification of tropical trees predict population dynamics after disturbance? Journal of Vegetation Science 19: 209–220.

[28] Gourlet-Fleury S, Guehl J, Laroussinie O (2004) Ecology and Management of a Neotropical Rainforest - Lessons drawn from Paracou, a long-term experimental research site in French Guiana. Paris: Elsevier.

[29] Cochran WG (1977) Sampling Techniques, 3rd Edition. Wiley, 448 pp.

[30] Sheil D, Burslem DFRP (2003) Disturbing hypotheses in tropical forests. Trends in Ecology and Evolution 18: 18–26.

[31] Cornelissen JHC, Lavorel S, Garnier E, Diaz S, Buchmann N, et al (2003) A handbook of protocols for standardised and easy measurement of plant functional traits worldwide. Australian Journal of Botany 51: 335–380.

[32] Baraloto C, Timothy Paine CE, Patiño S, Bonal D, Hérault B, et al (2010) Functional trait variation and sampling strategies in species-rich plant communities. Functional Ecology 24: 208–216.

[33] King DA, Davies SJ, Tan S, Noor NSM (2006) The role of wood density and stem support costs in the growth and mortality of tropical trees. Journal of Ecology 94: 670–680.

[34] Heckerman D, Geiger D, Chickering DM (1995) Learning bayesian networks: The combination of knowledge and statistical data. Machine Learning 20: 197–243.

[35] Pearl J (1998) Bayesian networks. Technical Report 980002. URL citeseer.ist.psu.edu/article/pearl98bayesian.html

[36] Boettcher SG, Dethlefsen C (2003) deal: A package for learning bayesian networks. Journal of Statistical Software 8: 1–40.

[37] Shipley B (2009) Confirmatory path analysis in a generalized multilevel context. Ecology 90: 363–368. 19323220 10.1890/08-1034.1

[38] Shipley B, Lechowicz M (2000) The functional co-ordination of leaf morphology, nitrogen concentration, and gas exchange in 40 wetland species. Ecoscience 7: 183–194.

[39] R Development Core Team (2009) R: A Language and Environment for Statistical Computing. R Foundation for Statistical Computing, Vienna, Austria. URL http://www.R-project.org. ISBN 3-900051-07-0.

[40] Herault B, Ouallet J, Blanc L, Wagner F, Baraloto C (2010) Growth responses of neotropical trees to logging gaps. Journal of Applied Ecology 47: 821–831.

[41] Sist P, Nguyen-Thé N (2002) Logging damage and the subsequent dynamics of a dipterocarp forest in east kalimantan (1990-1996). Forest Ecology and Management 165: 85–103.

[42] Kammesheidt L, Köhler P, Huth A (2001) Sustainable timber harvesting in venezuela: a modelling approach. Journal of Applied Ecology 38: 756–770.

[43] Peñuelas J, Poulter B, Sardans J, Ciais P, van der Velde M, et al (2013) Human-induced nitrogen–phosphorus imbalances alter natural and managed ecosystems across the globe. Nature Communications 4. 10.1038/ncomms3934 24343268

[44] Raaimakers D, Boot RGA, Dijkstra P, Pot S (1995) Photosynthetic rates in relation to leaf phosphorus content in pioneer versus climax tropical rainforest trees. Oecologia 102: 120–125. 28306816 10.1007/BF00333319

[45] Güsewell S (2004) N : P ratios in terrestrial plants: variation and functional significance. New Phytologist 164: 243–266. 10.1111/j.1469-8137.2004.01192.x 33873556

[46] Mercado LM, Patiño S, Domingues TF, Fyllas NM, Weedon GP, et al (2011) Variations in amazon forest productivity correlated with foliar nutrients and modelled rates of photosynthetic carbon supply. Philosophical Transactions of the Royal Society B: Biological Sciences 366: 3316–3329. 10.1098/rstb.2011.0045 PMC3179632 22006971

[47] Nascimento HE, Laurance WF, Condit R, Laurance SG, D'Angelo S, et al (2005) Demographic and life-history correlates for amazonian trees. Journal of Vegetation Science 16: 625–634.

[48] Poorter L, Bongers L, Bongers F (2006) Architecture of 54 moist-forest tree species: traits, trade-offs, and functional groups. Ecology 87: 1289–1301. 16761607 10.1890/0012-9658(2006)87[1289:aomtst]2.0.co;2

[49] Rüger N, Williams-Linera G, Kissling WD, Huth A (2008) Long-term impacts of fuelwood extraction on a tropical montane cloud forest. Ecosystems 11: 868–881.

[50] Anten NPR, Schieving F (2010) The role of wood mass density and mechanical constraints in the economy of tree architecture. The American Naturalist 175: 250–260. 10.1086/649581 20028240

[51] Markesteijn L, Poorter L, Paz H, Sack L, Bongers F (2011) Ecological differentiation in xylem cavitation resistance is associated with stem and leaf structural traits. Plant, Cell & Environment 34: 137–148. 10.1111/j.1365-3040.2010.02231.x 20946587

[52] Santiago LS, Goldstein G, Meinzer FC, Fisher JB, Machado K, et al (2004) Leaf photosynthetic traits scale with hydraulic conductivity and wood density in panamanian forest canopy trees. Oecologia 140: 543–550. 15232729 10.1007/s00442-004-1624-1

[53] Hérault B, Bachelot B, Poorter L, Rossi V, Bongers F, et al (2011) Functional traits shape ontogenetic growth trajectories of rain forest tree species. Journal of Ecology 99: 1431–1440.

[54] Geritz SA (1995) Evolutionarily stable seed polymorphism and small-scale spatial variation in seedling density. American Naturalist : 685–707.

[55] Kraft NJB, Metz MR, Condit RS, Chave J (2010) The relationship between wood density and mortality in a global tropical forest data set. New Phytologist 188: 1124–1136. 21058950 10.1111/j.1469-8137.2010.03444.x

[56] Aubry-Kientz M, Hérault B, Ayotte-Trépanier C, Baraloto C, Rossi V (2013) Toward trait-based mortality models for tropical forests. PLoS ONE 8: e63678. 23675500 10.1371/journal.pone.0063678 PMC3652824

[57] Russo SE, Brown P, Tan S, Davies SJ (2008) Interspecific demographic trade-offs and soil-related habitat associations of tree species along resource gradients. Journal of Ecology 96: 192–203.

[58] Gourlet-Fleury S, Rossi V, Rejou-Mechain M, Freycon V, Fayolle A, et al (2011) Environmental filtering of dense-wooded species controls above-ground biomass stored in african moist forests. Journal of Ecology 99: 981–990.

[59] Farquhar GD, Ehleringer JR, Hubick KT (1989) Carbon isotope discrimination and photosynthesis. Annual Review of Plant Physiology and Plant Molecular Biology

[60] Wagner F, Rossi V, Stahl C, Bonal D, Hérault B (2012) Water availability is the main climate driver of neotropical tree growth. PLoS ONE 7: e34074. 22506012 10.1371/journal.pone.0034074 PMC3323616

[61] Stahl C, Hérault B, Rossi V, Burban B, Bréchet C, et al (2013) Depth of soil water uptake by tropical rainforest trees during dry periods: does tree dimension matter? Oecologia 173: 1191–1201. 23852028 10.1007/s00442-013-2724-6

[62] Reich PB, Walters MB, Ellsworth DS (1997) From tropics to tundra: global convergence in plant functioning. Proceedings of the National Academy of Sciences 94: 13730–13734. 10.1073/pnas.94.25.13730 PMC28374 9391094

[63] Sparks JP, Ehleringer JR (1997) Leaf carbon isotope discrimination and nitrogen content for riparian trees along elevational transects. Oecologia 109: 362–367. 28307532 10.1007/s004420050094

[64] Schulze ED, Williams RJ, Farquhar GD, Schulze W, Langridge J, et al (1998) Carbon and nitrogen isotope discrimination and nitrogen nutrition of trees along a rainfall gradient in northern australia. Aust J Plant Physiol 25: 413–425.

[65] Lamont BB, Groom PK, Cowling RM (2002) High leaf mass per area of related species assemblages may reflect low rainfall and carbon isotope discrimination rather than low phosphorus and nitrogen concentrations. Functional Ecology 16: 403–412.

[66] Cernusak LA, Ubierna N, Winter K, Holtum JAM, Marshall JD, et al (2013) Environmental and physiological determinants of carbon isotope discrimination in terrestrial plants. New Phytologist 200: 950–965. 23902460 10.1111/nph.12423

[67] Vitousek PM, Field CB, Matson PA (1990) Variation in foliar *δ* ^13^C in Hawaiian *Metrosideros polymorpha*: a case of internal resistance? Oecologia 84: 362–370. 28313026 10.1007/BF00329760

[68] Hultine KR, Marshall JD (2000) Altitude trends in conifer leaf morphology and stable carbon isotope composition. Oecologia 123: 32–40. 28308741 10.1007/s004420050986

[69] Meinzer FC, Rundel PW, Goldstein G, Sharifi MR (1992) Carbon isotope composition in relation to leaf gas exchange and environmental conditions in hawaiian metrosideros polymorpha populations. Oecologia 91: 305–311. 28313536 10.1007/BF00317617

[70] Poorter H, Farquhar G (1994) Transpiration, intracellular carbon dioxide concentration and Carbon-Isotope discrimination of 24 wild species differing in relative growth rate. Functional Plant Biol 21: 507–516.

[71] Donovan LA, Ehleringer JR (1994) Carbon isotope discrimination, water-use efficiency, growth, and mortality in a natural shrub population. Oecologia 100: 347–354. 28307020 10.1007/BF00316964

[72] Koch GW, Sillett SC, Jennings GM, Davis SD (2004) The limits to tree height. Nature 428: 851–854. 15103376 10.1038/nature02417

[73] Ryan MG, Yoder BJ (1997) Hydraulic limits to tree height and tree growth. BioScience 47: 235–242.

[74] Reich PB, Wright IJ, Cavender-Bares J, Craine JM, Oleksyn J, et al (2003) The evolution of plant functional variation: Traits, spectra, and strategies. International Journal of Plant Sciences 164: S143–S164.

[75] Hérault B (2007) Reconciling niche and neutrality through the emergent group approach. Perspectives in Plant Ecology, Evolution and Systematics 9: 71–78.

[76] Baraloto C, Hérault B, Paine CET, Massot H, Blanc L, et al (2012) Contrasting taxonomic and functional responses of a tropical tree community to selective logging. Journal of Applied Ecology 49: 861–870.

