# Supplementary figures and images for: Functional Traits Help Predict Post-Disturbance Demography of Tropical Trees
Source: PLoS One. 2014 Sep 16;9(9):e105022. doi: 10.1371/journal.pone.0105022 (PMC4165593; doi:10.1371/journal.pone.0105022)

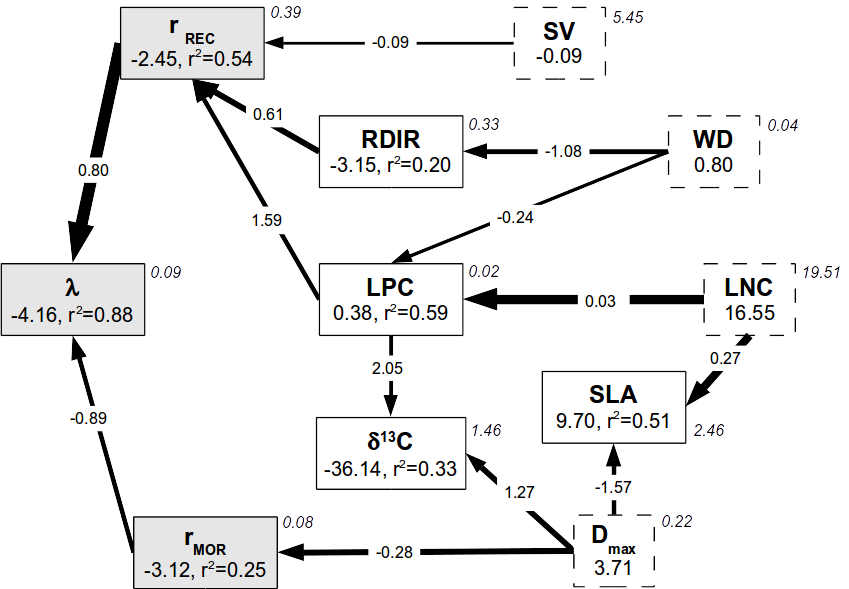

Supplement: Figure S1 — Bayesian Network resulting from the addition of species growth amongst the set of studied variables. (TIF) [file pone.0105022.s001.tif]
