# Supplementary material for: Functional Traits Help Predict Post-Disturbance Demography of Tropical Trees
Source: PLoS One. 2014 Sep 16;9(9):e105022. doi: 10.1371/journal.pone.0105022 (PMC4165593; doi:10.1371/journal.pone.0105022)
Supplement: Appendix S1 — Tables S1-S4. Table S1. Demographic and dynamics information on the 53 tropical tree species of the study. Table S2. Functional traits data for the tree species of the study. Table S3. Statistical summary of variables distributions and transformations. Table S4. Conditional independences derived from the DAG presented in main text. (PDF) [file pone.0105022.s002.pdf]

Supporting information

Table S1. Population data for the 53 studied species regarding the study period (1992–2003, see main text for definitions): N_{1992}, population size at the start of the study period, λ, population change rate, r_{MOR}: mortality rate, r_{REC}: recruitment rate, RDIR: relative diameter increase rate. The species are classified in four functional groups based on potential size and light requirement: small- to medium-sized heavy shade-tolerant species (HT, $n = 12$ species), medium-sized tolerant species (T, $n = 14$), emergent mid-tolerant species (MT, $n = 10$), heliophilous species from small to canopy species, including three true pioneers (H, $n = 13$).

Label	Species name	Botanical family	Group	N_{1992}	λ	r_{MOR}	r_{REC}	RDIR
Ac	*Andira coriacea*	Papilionaceae	MT	42	0.008	0.009	0.018	0.032
Ag	*Apeiba glabra*	Tiliaceae	H	36	0.028	0.010	0.038	0.026
Bap	*Balizia pedicellaris*	Mimosaceae	H	19	0.056	0.008	0.054	0.086
Bop	*Bocoa prouacensis*	Caesalpiniaceae	HT	388	−0.003	0.012	0.007	0.027
Cag	*Caryocar glabrum*	Caryocaraceae	MT	35	0.017	0.002	0.022	0.024
Cap	*Carapa procera*	Meliaceae	H	225	0.016	0.021	0.034	0.037
Cas	*Chaetocarpus schomburgkianus*	Euphorbiaceae	T	101	−0.003	0.012	0.009	0.027
Cf	*Catostemma fragrans*	Bombacaceae	HT	130	0.014	0.011	0.024	0.029
Chp	*Chrysophyllum prieurii*	Sapotaceae	T	97	0.005	0.007	0.012	0.028
Chs	*Chrysophyllum sanguinolentum*	Sapotaceae	T	122	0.001	0.014	0.014	0.041
Cm	*Couratari multiflora*	Lecythidaceae	T	144	0.013	0.008	0.017	0.027
Cng	*Conceveiba guianensis*	Euphorbiaceae	H	68	0.027	0.007	0.037	0.032
Cug	*Couma guianensis*	Apocynaceae	T	26	0.034	0.003	0.033	0.022
Db	*Dendrobangia boliviana*	Icacinaceae	T	31	0.034	0.007	0.039	0.052
Dg	*Dicorynia guianensis*	Caesalpiniaceae	MT	211	0.015	0.004	0.017	0.032
Ef	*Eperua falcata*	Caesalpiniaceae	MT	669	0.011	0.003	0.013	0.029
Eg	*Eperua grandiflora*	Caesalpiniaceae	MT	180	0.016	0.003	0.018	0.045
Gg	*Goupia glabra*	Celastraceae	H	55	0.048	0.009	0.058	0.036

Table S1. *(continued)*

Label	Species name	Botanical family	Group	N_{1992}	λ	r_{MOR}	r_{REC}	RDID
Gh	*Gustavia hexapetala*	Lecythidaceae	HT	183	0.013	0.008	0.019	0.029
Gm	*Garcinia madruno*	Clusiaceae	HT	96	0.010	0.013	0.025	0.029
Ih	*Iryanthera hostmannii*	Myristicaceae	HT	90	−0.004	0.009	0.007	0.027
Ip	*Inga pezizifera*	Mimosaceae	H	201	0.103	0.008	0.093	0.047
Is	*Iryanthera sagotiana*	Myristicaceae	HT	242	0.000	0.015	0.015	0.033
Jac	*Jacaranda copaia*	Bignoniaceae	H	69	0.123	0.016	0.110	0.044
La	*Licania alba*	Chrysobalanaceae	T	644	0.003	0.009	0.012	0.032
Lap	*Laetia procera*	Flacourtiaceae	H	22	0.113	0.009	0.104	0.035
Lep	*Lecythis persistens*	Lecythidaceae	HT	1330	0.005	0.007	0.011	0.029
Lh	*Licania heteromorpha*	Chrysobalanaceae	T	411	0.004	0.018	0.023	0.031
Mb	*Manilkara bidentata*	Sapotaceae	T	41	0.014	0.004	0.017	0.021
Mrc	*Moronobea coccinea*	Clusiaceae	MT	100	0.003	0.009	0.013	0.033
Muc	*Mouriri crassifolia*	Melastomataceae	T	139	0.006	0.017	0.024	0.030
Oa	*Oxandra asbeckii*	Annonaceae	HT	701	0.014	0.011	0.026	0.032
Pc	*Pradosia cochlearia*	Sapotaceae	T	300	0.006	0.014	0.016	0.032
Pi	*Platonia insignis*	Clusiaceae	MT	25	0.010	0.000	0.009	0.018
Pl	*Posoqueria latifolia*	Rubiaceae	HT	119	0.007	0.009	0.017	0.032
Ps	*Pogonophora schomburgkiana*	Euphorbiaceae	HT	647	−0.010	0.024	0.015	0.032
Qr	*Qualea rosea*	Vochysiaceae	MT	155	0.039	0.004	0.043	0.030
Rs	*Recordoxylon speciosum*	Caesalpiniaceae	MT	132	0.006	0.013	0.018	0.043
Sc	*Simaba cedron*	Simaroubaceae	HT	202	0.018	0.003	0.019	0.025
Sd	*Schefflera decaphylla*	Araliaceae	H	19	0.249	0.004	0.151	0.102

Table S1. *(continued)*

Label	Species name	Botanical family	Group	N_{1992}	λ	r_{MOR}	r_{REC}	RDID
Sg	*Sandwithia guianensis*	Euphorbiaceae	HT	31	0.003	0.024	0.026	0.032
Spa	*Swartzia panacoco*	Caesalpiniaceae	T	34	0.012	0.007	0.018	0.030
Spo	*Swartzia polyphylla*	Caesalpiniaceae	MT	38	0.040	0.003	0.039	0.034
Sr	*Sextonia rubra*	Lauraceae	MT	48	0.002	0.010	0.010	0.025
Stp	*Sterculia pruriens*	Sterculiaceae	H	147	0.107	0.006	0.093	0.051
Swg	*Swartzia guianensis*	Caesalpiniaceae	HT	69	0.016	0.018	0.031	0.031
Sy	*Symphonia sp1*	Clusiaceae	MT	357	0.011	0.011	0.021	0.056
Tc	*Tapura capitulifera*	Dichapetalaceae	T	107	−0.003	0.007	0.004	0.028
Tg	*Tapirira guianensis*	Anacardiaceae	H	57	0.228	0.005	0.148	0.112
Tm	*Tachigali melinonii*	Caesalpiniaceae	H	42	0.075	0.005	0.071	0.097
Ts	*Theobroma subincanum*	Sterculiaceae	HT	89	−0.003	0.011	0.009	0.042
Va	*Vouacapoua americana*	Caesalpiniaceae	T	251	0.005	0.005	0.008	0.027
Vm	*Virola michelii*	Myristicaceae	H	97	0.057	0.006	0.062	0.032

Table S2. Trait data for the 53 studied species : specific leaf area (SLA, $m^2.kg^{-1}$), leaf nitrogen concentration (LNC, ‰), leaf phosphorus concentration (LPC, ‰), carbon isotope composition ()δ^{13}, ‰), wood density (WD, $g.cm^{-3}$), maximal diameter (D_m, cm), seed volume (SV, cm^3). Label indicates species (see Table S1).

Label	SLA	LPC	LNC	WD	SV	δ^{13}C	D_{max}
Ac	6.51	0.61	17.36	0.85	27.61	−28.41	59
Ag	10.37	0.53	13.59	0.38	0.00	−30.55	37
Bap	6.96	0.59	17.20	0.61	0.13	−28.45	96
Bop	7.59	0.55	20.66	1.23	1.06	−30.02	42
Cag	8.97	0.78	15.30	0.82	36.82	−28.80	71
Cap	6.46	0.81	14.06	0.70	40.36	−29.80	39
Cas	4.81	0.39	9.08	1.13	0.05	−31.18	47
Cf	7.25	0.52	12.41	0.89	14.70	−31.92	31
Chp	7.59	0.88	22.75	1.27	2.45	−28.72	43
Chs	6.95	0.52	11.82	0.73	0.66	−29.32	46
Cm	8.22	0.70	20.33	0.61	0.07	−27.67	45
Cng	10.77	0.89	18.64	0.68	0.38	−29.10	28
Cug	8.48	0.66	13.55	0.58	0.11	−28.53	38
Db	10.18	0.61	18.28	0.80	0.34	−32.05	45
Dg	7.20	0.72	18.52	0.76	0.30	−28.05	61
Ef	8.58	0.74	15.67	0.88	5.49	−28.78	63
Eg	6.41	0.66	12.81	0.95	60.30	−28.70	65
Gg	11.01	0.62	15.47	0.84	0.00	−29.48	65
Gh	11.36	0.64	17.76	0.85	1.02	−32.00	20
Gm	7.98	0.38	11.52	0.75	1.00	−32.03	26
Ih	8.89	0.61	15.17	0.75	0.81	−34.15	24

Table S2. *(continued)*

alpcod	sla	lpc	lnc	wd	sevol	dc13	dmax
Ip	12.97	1.10	30.46	0.66	0.17	−30.95	37
Is	9.29	0.75	16.48	0.77	1.91	−31.43	28
Jac	9.72	1.00	20.81	0.47	0.75	−28.61	38
La	5.41	0.27	9.38	1.06	5.87	−31.87	49
Lap	7.80	0.89	19.70	0.84	0.01	−29.62	49
Lep	6.20	0.45	15.59	0.72	3.30	−32.75	25
Lh	6.61	0.42	12.51	1.03	2.00	−30.85	36
Mb	4.77	0.43	11.91	1.10	1.59	−29.27	55
Mrc	9.27	0.46	12.74	0.93	2.95	−29.66	45
Muc	6.81	0.39	11.02	1.10	0.14	−31.56	35
Oa	10.22	0.48	18.35	0.90	0.54	−31.78	15
Pc	8.15	0.42	10.23	0.96	0.95	−30.25	67
Pi	5.52	0.44	12.39	0.84	13.36	−27.62	69
Pl	8.18	0.38	12.18	0.71	2.98	−31.27	21
Ps	9.97	0.42	13.70	0.97	0.01	−30.43	22
Qr	7.47	0.67	13.83	0.71	1.16	−29.06	71
Rs	11.23	0.77	19.87	1.04	0.24	−29.18	52
Sc	9.01	0.81	22.56	0.60	40.51	−27.66	18
Sd	7.63	1.11	18.80	0.49	0.04	−28.71	23

Table S2. *(continued)*

alpcod	sla	lpc	lnc	wd	sevol	dc13	dmax
Sg	15.80	0.50	19.29	0.86	0.10	−30.53	15
Spa	7.19	0.58	20.61	1.11	3.00	−29.43	42
Spo	8.46	0.76	22.70	1.00	44.91	−30.37	150
Sr	7.21	0.60	14.01	0.64	1.50	−29.93	78
Stp	7.34	0.79	15.64	0.65	1.65	−30.60	49
Swg	12.81	0.80	27.25	0.90	1.13	−31.22	19
Sy	7.74	0.49	15.69	0.67	1.75	−30.22	42
Tc	3.84	0.51	13.28	0.68	0.47	−29.08	70
Tg	7.10	0.71	14.50	0.50	0.26	−31.01	34
Tm	11.99	0.79	27.89	0.49	1.31	−29.09	66
Ts	9.19	0.71	15.11	0.55	1.68	−31.77	25
Va	10.68	0.96	19.72	0.90	29.73	−31.65	59
Vm	8.51	0.67	17.00	0.49	2.48	−32.17	38

Table S3. Summary of variables distribution indicating the mean, standard deviation, minimal and maximal values ($n = 53$ species).
Offset : indicates the offset value in the transformation $\log(x + o)$, where x is the transformed variable and o is the offset chosen to maximise the normality of the variable after transformation (Shapiro-Francia test, function *sf.test* from R package *normtest*). No value indicates untransformed variables. References: 1, CIRAD; 2: [1]; 3: [2]; 4: [3]; 5: [4]; 6: [5]; 7: [6]; * indicates this study.

Variable	Label	Offset	Unit	Mean	S.Dev.	Min.	Max.	Reference
Demography after disturbance								
Population change rate	λ	0.013	$\%.yr^{-1}$	-3.55	0.86	-5.90	-1.34	*
Recruitment rate	r_{REC}	-0.002	%	-3.87	0.92	-6.23	-1.90	*
Mortality rate	r_{MOR}	0.007	%	-4.17	0.32	-4.96	-3.48	*
Traits in undisturbed conditions								
Specific leaf area	SLA		$m^2.kg^{-1}$	8.43	2.25	3.84	15.80	*
Leaf nitrogen concentration	LNC		‰	16.55	4.58	9.08	30.46	*
Leaf phosphorus concentration	LPC		‰	0.64	0.19	0.27	1.11	*
Carbon isotope composition	$\delta^{13}C$		‰	-30.14	1.48	-34.15	-27.62	*
Wood density	WD		$g.cm^{-3}$	0.80	0.21	0.38	1.27	1–3
Maximal diameter	D_m	0	cm	3.71	0.48	2.71	5.01	*
Seed volume	SV	0	cm^3	-0.09	2.42	-6.91	4.10	*, 4–7

References

1. Gerard J, Miller R, ter Welle B (1996) Major timber trees of Guyana, timber characteristics and utilization, volume 461195687 of *Tropenbos series – 15*. CAB International Press.

2. ter Steege H, Hammond DS (2001) Character convergence, diversity, and disturbance in tropical rain forest in guyana. Ecology 82: 3197-3212.

3. Sabatier D (1983) Fructification et dissémination en forêt guyanaise - L'exemple de quelques espèces ligneuses. Phd, Université des Sciences et Techniques du Languedoc.

4. Roosmalen Mv (1985) Fruits of the Guianan flora. Utrecht: Institute of systematic Botany University.

5. Collinet F (1997) Essai de regroupement des principales espèces structurantes d'une forêt dense humide d'après l'analyse de leur répartition spatiale (Forêt de Paracou - Guyane). Ph.D. thesis, Université Claude Bernard - Lyon 1.

6. Mori S, Cremers G, Gracie C, de Granville J, Heald S, et al. (2002) Guide to the vascular plants of central French Guiana. Part 2. Dicotyledons. Memoirs-New York Botanical Garden .

Table S4. Basis set of independent relationships [37] that derive from the Directed Acyclic Graph obtained by Bayesian Network analysis. The structure of the DAG implies a basis set of conditional and unconditional independencies ($\perp$) from which all others can be deduced. These relationships were tested *a posteriori*, using Spearman's correlation coefficient between the variables in the first column, either conditioned on the set of variables in second column, or unconditoned. r_{spea} indicates the corresponding value of the coefficient and p the p-value for the test. P-values were adjusted for multiple tests. All tests were not significant, indicating expected statistical independency.

	**Variables**	**Conditioning set**	r_{spea}	p
1.	WD $\perp$ SV		0.20	0.58
2.	WD $\perp$ LNC		−0.14	0.64
3.	WD $\perp$ D_{max}		0.11	0.67
4.	WD $\perp$ SLA	LNC,D_{max}	−0.10	0.50
5.	WD $\perp$ $\delta^{13}C$	LPC,D_{max}	−0.11	0.64
6.	WD $\perp$ r_{MOR}	D_{max}	0.28	0.70
7.	WD $\perp$ λ	r_{REC},r_{MOR}	−0.14	0.67
8.	SV $\perp$ LNC		−0.04	0.81
9.	SV $\perp$ D_{max}		0.16	0.58
10.	SV $\perp$ LPC	WD,LNC	0.18	0.58
11.	SV $\perp$ SLA	LNC,D_{max}	−0.25	0.50
12.	SV $\perp$ $\delta^{13}C$	LPC,D_{max}	−0.12	0.80
13.	SV $\perp$ r_{MOR}	D_{max}	−0.24	0.50
14.	SV $\perp$ λ	r_{REC},r_{MOR}	0.06	0.67
15.	LNC $\perp$ D_{max}		−0.09	0.81
16.	LNC $\perp$ $\delta^{13}C$	LPC,D_{max}	0.08	0.70
17.	LNC $\perp$ r_{REC}	WD,SV,LPC	0.05	0.50
18.	LNC $\perp$ r_{MOR}	D_{max}	−0.22	0.58
19.	LNC $\perp$ λ	r_{REC},r_{MOR}	0.16	0.74
20.	LPC $\perp$ D_{max}	WD,LNC	0.25	0.50
21.	LPC $\perp$ SLA	WD,LNC,D_{max}	0.07	0.58
22.	LPC $\perp$ r_{MOR}	WD,LNC,D_{max}	−0.17	0.50
23.	LPC $\perp$ λ	WD,LNC,r_{REC},r_{MOR}	0.26	0.75
24.	$D_{max}$$\perp$ λ	r_{REC},r_{MOR}	0.18	0.81
25.	SLA $\perp$ $\delta^{13}C$	LNC,D_{max},LPC	−0.24	0.81
26.	$r_{REC}$$\perp$ D_{max}	WD,SV,LPC	−0.04	0.81
27.	$r_{REC}$$\perp$ r_{MOR}	WD,SV,LPC,D_{max}	−0.04	0.73
28.	$r_{REC}$$\perp$ SLA	WD,SV,LPC,LNC,D_{max}	0.03	0.58
29.	$r_{REC}$$\perp$ $\delta^{13}C$	WD,SV,LPC,D_{max}	−0.09	0.67
30.	$r_{MOR}$$\perp$ SLA	D_{max},LNC	0.12	0.58
31.	$r_{MOR}$$\perp$ $\delta^{13}C$	D_{max},LPC	−0.17	0.67
32.	λ $\perp$ SLA	r_{REC},r_{MOR},LNC,D_{max}	−0.12	0.70
33.	λ $\perp$ $\delta^{13}C$	r_{REC},r_{MOR},LPC,D_{max}	0.10	0.50

Figure S1. Bayesian Network analysis with species growth potential included (RDID: relative diameter increase rate) as the 95th percentile of the distribution of change rate in relative diameter increment $\frac{\Delta \ln(D_t)}{\Delta t}$, D_t being the DBH at year t, and Δt taken here between 2000 and 2003). See Figure 2 for comparison and its legend for explanations. Grey (resp. white) indicates variables measured after disturbance (in control conditions). Dashed boxes indicate variables having no parent in the network: SV, WD, LNC and D_{max}. Numbers indicate the posterior estimates of the model parameters: the intercept $\hat{\alpha}$ and the proportion of variance explained by the parents (r^2) are given in boxes, the regression coefficients ($\hat{\beta}$) are shown on arrows. Arrow width is proportional to the corresponding standardized regression coefficient. Italic numbers are estimates of the residual variance ($\hat{\sigma^2}$).
